# Tumor Necrosis Factor Alpha Inhibits L-Type Ca^2+^ Channels in Sensitized Guinea Pig Airway Smooth Muscle through ERK 1/2 Pathway

**DOI:** 10.1155/2016/5972302

**Published:** 2016-06-30

**Authors:** Jorge Reyes-García, Edgar Flores-Soto, Héctor Solís-Chagoyán, Bettina Sommer, Verónica Díaz-Hernández, Luz María García-Hernández, Luis M. Montaño

**Affiliations:** ^1^Departamento de Farmacología, Facultad de Medicina, Universidad Nacional Autónoma de México, 04510 Ciudad de México, DF, Mexico; ^2^Posgrado en Ciencias Biológicas, Universidad Nacional Autónoma de México, 04510 Ciudad de México, DF, Mexico; ^3^Laboratorio de Neurofarmacología, Instituto Nacional de Psiquiatría Ramón de la Fuente Muñiz, 14370 Ciudad de México, DF, Mexico; ^4^Departamento de Investigación en Hiperreactividad Bronquial, Instituto Nacional de Enfermedades Respiratorias, 14080 Ciudad de México, DF, Mexico; ^5^Departamento de Embriología, Facultad de Medicina, Universidad Nacional Autónoma de México, 04510 Ciudad de México, DF, Mexico

## Abstract

Tumor necrosis factor alpha (TNF-*α*) is a potent proinflammatory cytokine that plays a significant role in the pathogenesis of asthma by inducing hyperresponsiveness and airway remodeling. TNF-*α* diminishes the L-type voltage dependent Ca^2+^ channel (L-VDCC) current in cardiac myocytes, an observation that seems paradoxical. In guinea pig sensitized tracheas KCl responses were lower than in control tissues. Serum from sensitized animals (Ser-S) induced the same phenomenon. In tracheal myocytes from nonsensitized (NS) and sensitized (S) guinea pigs, an L-VDCC current (ICa) was observed and diminished by Ser-S. The same decrease was detected in NS myocytes incubated with TNF-*α*, pointing out that this cytokine might be present in Ser-S. We observed that a small-molecule inhibitor of TNF-*α* (SMI-TNF) and a TNF-*α* receptor 1 (TNFR1) antagonist (WP9QY) reversed ICa decrease induced by Ser-S in NS myocytes, confirming the former hypothesis. U0126 (a blocker of ERK 1/2 kinase) also reverted the decrease in ICa. Neither cycloheximide (a protein synthesis inhibitor) nor actinomycin D (a transcription inhibitor) showed any effect on the TNF-*α*-induced ICa reduction. We found that Ca_V_1.2 and Ca_V_1.3 mRNA and proteins were expressed in tracheal myocytes and that sensitization did not modify them. In cardiac myocytes, ERK 1/2 phosphorylates two sites of the L-VDCC, augmenting or decreasing ICa; we postulate that, in guinea pig tracheal smooth muscle, TNF-*α* diminishes ICa probably by phosphorylating the L-VDCC site that reduces its activity through the ERK1/2 MAP kinase pathway.

## 1. Introduction

Tumor necrosis factor alpha (TNF-*α*) has been characterized as a potent proinflammatory cytokine that plays a significant role in the pathogenesis of asthma [1–3]. Furthermore, in the sputum and in the bronchoalveolar lavage fluid of asthmatic patients, this cytokine concentration was increased [4, 5]. It has also been reported that, in plasma and bronchoalveolar lavage fluid from sensitized guinea pigs, TNF-*α* was significantly augmented [6]. Multiple sources point out that, in the airways, TNF-*α* produces many different effects: it enhances airway smooth muscle contractile response to different agonists (carbachol, histamine, and bradykinin), it augments agonist-induced Ca^2+^ transients, it diminishes the relaxation induced by isoproterenol, and it favors airway remodeling. All these events contribute to the airway hyperresponsiveness development [7–10].

It is well known that L-type voltage dependent Ca^2+^ channel (L-VDCC) by itself plays a minor role in the agonists-induced airway smooth muscle contraction [11, 12]. Recently, we confirmed that this channel mainly provides extracellular Ca^2+^ to refill the sarcoplasmic reticulum (SR), probably favoring agonists-induced contractile responses [12]. However, in the eighties, its participation in the pathogenesis of asthma was considered controversial since the use of L-VDCC blockers showed great variability in its effects during the treatment of this disease [13], and this inconsistency was never totally clarified. Nowadays, many aspects of inflammation have been thoroughly investigated and it is known that proinflammatory cytokines such as TNF-*α* alter L-VDCC function in rat cardiac myocytes. In this regard, it has been demonstrated that it reduces, in a reversible manner, the L-VDCC current (ICa) in these cells through the activation of TNF-*α* receptor 1 (TNFR1) [14, 15]. This TNF-*α* induced alteration of the L-VDCC function might be happening in airway smooth muscle and could explain the great variability of the L-VDCC blockers effects seen in asthmatic patients.

TNF-*α* responses in airway smooth muscle have been documented to be mediated by two receptor subtypes, TNFR1 and TNFR2 (also known as p55TNFR and p75TNFR) [16]. TNFR1 activation has been related to augmented agonist-induced Ca^2+^ transients, airway smooth muscle proliferation through modulation of cell mitogenesis [7], upregulation of G proteins (G_i_, G_q_) [17], and molecules associated with sarcoplasmic reticulum (SR) Ca^2+^ handling such as CD38/cyclic ADP-ribose [18]; all these effects promote airway hyperresponsiveness [19]. Additionally, by activating TNFR1 in airway smooth muscle, TNF-*α* triggers extracellular signal-regulated kinase (ERK) and p38 mitogen-activated protein kinases (MAPKs) signaling pathway and transcription factors to turn on a variety of genes (interleukins) that mediate inflammatory and immune responses [16]. Meanwhile, through TNFR2, this cytokine activates c-Jun N-terminal kinase (JNK), but not MAPK or p38 MAPKs signaling pathways, and its function is linked to enhance apoptotic cell death [20].

For a long time, in airway smooth muscle, the L-VDCC was characterized through pharmacological and electrophysiological methods [11, 21]. Nevertheless, Du et al. [22] claimed that, using molecular assays, they found all subunits of this channel (Ca_V_1.1, Ca_V_1.2, Ca_V_1.3, and Ca_V_1.4) in the rat bronchial smooth muscle.

Therefore, the aim of the present work was to explore the role of TNF-*α* on the functionality of the guinea pig airway smooth muscle L-VDCC and define the signaling pathway induced by this cytokine that could be acting on the channel. Additionally, we explored which subunits of the L-VDCC are present in this tissue and if they were modified by sensitization.

## 2. Material and Methods

### 2.1. Experimental Animals

Hartley male guinea pigs weighing 400–600 g from our institutional animal facilities (filtered conditioned air, 21 ± 1°C, 50–70% humidity, sterilized bed) fed with Harlan® pellets and sterilized water were used. The experimental protocol was approved by the Scientific and Bioethics Committees of the Facultad de Medicina, Universidad Nacional Autónoma de México (061/215). The experimental protocol closely followed the Guiding Principles for the Care and Use of Vertebrate Animals in Research and Training published by the American Physiological Society. Mexican National Protection Laws on Animal Protection and the General Health Law Related to Health Research (NOM-062-Z00-1999) were also considered.

### 2.2. Sensitization Procedure

Male guinea pigs were sensitized as described elsewhere [23]. Briefly, at day 0, animals weighing ~250 g received an i.p. and s.c. administration of 500 *μ*g ovalbumin (OA) and 500 *μ*g Al(OH)_3_ in 0.5 mL saline (0.9% NaCl). At day 8, they were nebulized during 80 sec with 15 mg/mL OA in saline delivered by an ultrasonic nebulizer (model WH-200, Guangdong Yuehua Medical Instruments Factory Co., Ltd., China) and again at day 15 with 1 mg/mL OA in saline during 10 sec. All animals were studied at days 21–25 of sensitization.

### 2.3. Organ Baths

Nonsensitized (NS) and sensitized (S) guinea pigs were anesthetized with pentobarbital sodium (35 mg/kg, i.p.) and exsanguinated. Eight rings were obtained from tracheas cleaned of connective tissue, and each ring was hung in a 5 mL organ bath chamber containing Krebs solution (in mM): 118 NaCl, 25 NaHCO_3_, 4.6 KCl, 1.2 KH_2_PO_4_, 1.2 MgSO_4_, 11 glucose, and 2 CaCl_2_ at 37°C. A mixture of 5% CO_2_ and 95% oxygen was used to bubble the tissue continuously and maintain the pH at 7.4. To block prostanoids formation, indomethacin (1 *μ*M) was added to the Krebs solution. Tension developed by the tissue was registered by an isometric force transducer (model FT03; Grass Instruments, West Warwick, RI, USA) connected to a signal conditioner (CyberAmp 380, Axon Instruments, Foster City, CA, USA) and to an analog-to-digital interface (Digidata 1440A; Axon Instruments). Data were recorded and analyzed with an AxoScope version 10.2 software (Axon Instruments).

Tracheal rings were submitted to a resting tension of 1 g during 30 min. Afterwards they were stimulated three times with KCl (60 mM) to allow tissue conditioning and optimization of the contractile apparatus. The last response to KCl was compared between NS and S tracheal tissues. In another set of experiments, NS tracheal preparations were incubated during 60 min with serum from sensitized animals at different percentage concentrations (V/V, 1, 2.5, 5, and 10%). Afterwards, a cumulative KCl concentration-response curve was done (20, 40, and 60 mM). These responses were expressed as % of the third 60 mM KCl stimulation.

### 2.4. Patch Clamp Studies

Guinea pig tracheal smooth muscle from NS and S animals was dissected free of epithelium and connective tissue and placed in 5 mL Hanks solution containing 2 mg L-cysteine and 0.04 U/mL papain. The pH was always adjusted to 7.4 with 1 M NaHCO_3_ and tissues were then incubated for 10 min at 37°C. The tissues were washed with Leibovitz's solution to remove enzyme excess and afterwards placed in Hanks solution with 1 mg/mL collagenase type I and 0.5 mg/mL protease during 10 min at 37°C. Myocytes were gently dispersed by mechanical agitation until detached cells were observed. Leibovitz's solution was used again to stop enzymatic activity and cells were centrifuged at 600 rpm, 20°C during 5 min, and the supernatant was discarded. This procedure was repeated once.

Tracheal myocytes from NS or S animals were cultured as follows: the cell pellet was resuspended in minimum essential medium containing either 10% fetal bovine serum (FBS), 10% serum from no-sensitized (Ser-NS) animals, or 10% serum from sensitized animals (Ser-S), 2 mM L-glutamine, 10 U/mL penicillin, 10 *μ*g/mL streptomycin, and 15 mM glucose and plated on round cover slips coated with rat tail sterile collagen. Some myocytes cultures from NS animals containing FBS or Ser-S were added with tumor necrosis factor alpha (TNF-*α*, 20, 200, or 1000 *μ*g/L; these concentrations were used previously to block L-VDCC in rat cardiac myocytes [15]), a small-molecule inhibitor of TNF-*α* (SMI-TNF, 32 *μ*M; at this concentration, it promotes subunit disassembly of TNF-*α* and inhibits its activity [24]), the TNF-*α* receptor 1 (TNFR1) antagonist (WP9QY, 3.2 or 10 *μ*M [25]), or an inhibitor of ERK 1/2 (U0126, 5 *μ*M [26]). We used U0126 since it has been reported that TNF-*α* effects in airway smooth muscle are, in part, through ERK 1/2 MAPKs signaling pathway [26]. Cells were then cultured at 37°C in a 5% CO_2_ in oxygen during 48 h. In another set of experiments, NS myocytes were cultured with FBS during 24 h for them to adhere to the surface of the round cover slips. Afterwards, they were incubated with 1000 *μ*g/L TNF-*α* or TNF-*α* with the protein synthesis inhibitor cycloheximide 20 *μ*M [27] or TNF-*α* with the transcription inhibitor actinomycin D 3 *μ*M [28] during further 24 h.

Subsequently, myocytes on the cover glass were placed at the bottom of the 0.7 mL perfusion chamber and allowed to settle down. The chamber was perfused by gravity (~1.5–2.0 mL/min) with an external solution containing Ba^2+^ to replace Ca^2+^ as the inward charge carrier to measure Ca^2+^ currents and in mM 136 NaCl, 6 CsCl, 5 BaCl_2_, 11 glucose, 10 HEPES, and 0.1 niflumic acid, pH 7.4 adjusted with CsOH. All experiments were performed at room temperature (~21°C).

To record Ca^2+^ currents activated by depolarizing voltage steps (i.e., voltage clamp) through an Axopatch 200A amplifier (Axon Instruments, Foster City, CA, USA), the standard whole-cell configuration was used. Patch pipettes were made with 1B200F-6 glass (Word Precision Instruments, Sarasota, FL, USA) using a horizontal micropipette puller (P-87, Sutter Instruments Co, Novato, CA). Each pipette had a resistance ranging from 2 to 4 MΩ. The internal solution consisted of (mM) 130 CsCl, 2 MgCl_2_, 10 HEPES, 10 EGTA, 3.6 ATP disodium salt, and 1.9 GTP sodium salt, pH 7.3, adjusted with CsOH. Currents were filtered at 1–5 KHz, digitized (Digidata 1440A, Axon) at 10 KHz, stored, and analyzed in a computer through specialized software (pClamp v10.2, Axon).

Tracheal myocytes showed Ca^2+^ currents when subjected to series of conditioning hyperpolarizing and depolarizing pulses of potentials ranging from −60 to +50 mV in 10 mV increments from a holding potential of −60 mV during 100 ms, 1 Hz. Changes in the currents from the protocols described above were evaluated as maximal current peak to each voltage tested.

### 2.5. Total RNA Extraction and RT-PCR

Total RNA was purified using the RNeasy Mini Kit (Qiagen, CA, USA), to prevent DNA contamination; DNase Set digestion (Qiagen) was used and eluted with RNAse-free water. Concentration of total RNA was measured using a NanoDrop 2000 spectrophotometer (Thermo Scientific, Barrington, IL, USA). RNA purity was evaluated by the absorbance ratios at 260 : 230 nm and 260 : 280 nm, where a 1.8 ratio was suitable for proceeding with cDNA synthesis. Total RNA was reverse-transcribed to cDNA using transcriptor reverse transcriptase (Roche, Life Science, IN, USA), random primer (Invitrogen, Life Technologies, CA, USA), and 1 *μ*g of total RNA. The oligonucleotides were designed based on the sequences reported in the NCBI database. The oligonucleotide sequence, PCR melting temperature (MT), and accession number of the different subunits of the L-VDCC (Ca_V_1.1, Ca_V_1.2, Ca_V_1.3, and Ca_V_1.4) are shown in Table 1. Negative controls were prepared without template and with 50 ng of each total RNA extracted. The amplification conditions were 5 min at 94°C, followed by 1 min at 94°C, 1 min to MT (see Table 1) and 1 min at 72°C, and a final extension of 10 min. The reactions were performed by duplicate. The PCR products were electrophoresed in a 1.5% agarose gel with GelRed (Biotium, CA, USA). The PCR products corresponded to the estimated length. Amplicon identity was corroborated by sequencing. Images were digitized using Typhoon FLA 9500 laser scanner (GE Healthcare, CT, USA).

### 2.6. Double Immunofluorescence

Tracheal tissues were fixed in 4% paraformaldehyde in sodium phosphate buffer (PBS), overnight at 4°C, and dehydrated with ascendant series of ethanol until their inclusion in paraffin. The tissue blocks were cut in 10 *μ*m slices. Paraffin was removed by incubation in xylol, followed by graded alcohols. Heat induced antigen retrieval was performed by placing slides in a pressure cooker (Biocare Medical, CA, USA) in 1x Diva Decloaker (Biocare Medical). Slices were transferred into PBS and permeabilized with 0.05% Tween-20 in PBS. To block nonspecific binding to proteins, 10% horse serum was applied on the slices for two h. The slices were incubated with the primary antibodies to Ca_V_1.2 and Ca_V_1.3 (subunits of L-VDCC, Alomone Labs., Cat. numbers ACC-003 and ACC-311, resp., Jerusalem, Israel), both antibodies at a dilution 1 : 50, overnight at 4°C. The secondary antibody Alexa488 donkey anti-rabbit IgG (Life Technologies, CA, USA) was incubated (1 : 200) for 30 min. The slices were incubated with the next primary antibody, *α*-actin (Santa Cruz Biotechnology, Cat. number sc-58669, TX, USA), and Alexa Fluor 555 donkey anti-mouse (Life Technologies) 1 : 400 for 30 min, as the secondary antibody. Tissue sections were kept with fluorescence mounting medium Dako (Dako, North America Inc., CA, USA).

To determine the specificity of immunofluorescence, the antigens for Ca_V_1.2 and Ca_V_1.3 were used to saturate the primary antibody. The nuclei were counterstained with DAPI (Life Technologies). The immunofluorescences were observed using a fluorescence microscope (Model Eclipse Ni-U, Nikon, Japan). For display purposes, merged images were constructed in which Ca_V_1.2 and Ca_V_1.3 were green, *α*-actin red, and nuclei blue.

### 2.7. Drugs and Chemicals

Tumor necrosis factor alpha (TNF-*α*), U0126 ethanolate, an inhibitor of ERK 1/2 kinase (1,4-diamino-2,3-dicyano-1,4-bis-(0-amino-phenylmercapto)butadiene ethanolate), cycloheximide, actinomycin D, and indomethacin were purchased from Sigma Chem. Co. (St. Louis, MO, USA). TNF-*α* small-molecule inhibitor and WP9QY, an antagonist of the TNF-*α* receptor 1, were purchased from Calbiochem (Darmstadt, Germany).

### 2.8. Statistical Analysis

Data values obtained in organ baths and RT-PCR experiments were analyzed through nonpaired Student's *t*-test or one-way analysis of variance followed by Dunnett's multiple comparison tests, as well as the results for the patch clamp experiments. Along the paper and figures, data are expressed as mean ± SEM. In patch clamp experiments, each cell belongs to a different animal. Statistical significance was set at *p* < 0.05 bimarginally.

## 3. Results

### 3.1. Sensitization Reduced KCl Responses in Tracheal Smooth Muscle from Guinea Pig

**Figure 1:**
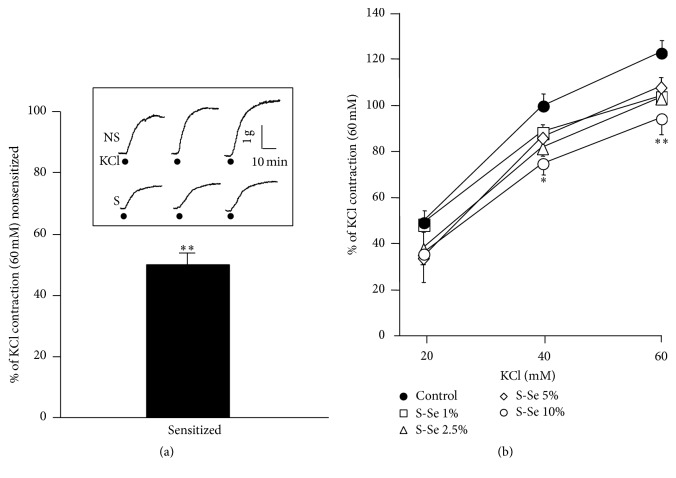
Sensitization diminished KCl-induced contraction in guinea pig tracheas. Responses to 3 consecutive stimulations of 60 mM KCl were higher in tracheal rings from nonsensitized guinea pigs (NS, *n* = 14) than in tissues from sensitized (S, *n* = 17) animals (inset). (a) The third KCl response from NS tissues was considered as 100%, and statistical difference was found when compared with the S group. (b) Nonsensitized tracheas, incubated with different percentage (V/V) concentrations of sensitized guinea pigs serum (Ser-S, *n* = 5, each), showed decreased responses to KCl (20–60 mM), reaching statistical significance only with the highest Ser-S concentration tested. ^*∗*^ *p* < 0.05, ^*∗∗*^ *p* < 0.01 compared with control group. Bars and symbols represent mean ± SEM.

In tracheal rings from NS guinea pigs, KCl induced a contraction that was significantly higher than the response observed in preparations from S animals (Figure 1(a)). NS tracheas, incubated with different percentage concentrations of sensitized guinea pigs serum (Ser-S, 1, 2.5, 5, and 10%, *n* = 5) showed decreased responses to cumulative concentrations of KCl (20, 40, and 60 mM), reaching statistical significance only with the highest Ser-S concentration tested (Figure 1(b)).

### 3.2. Possible Role of TNF-*α* in the Decrease of Ca^2+^ Currents Induced by Serum from Sensitized Animals in Tracheal Myocytes from Guinea Pig

**Figure 2:**
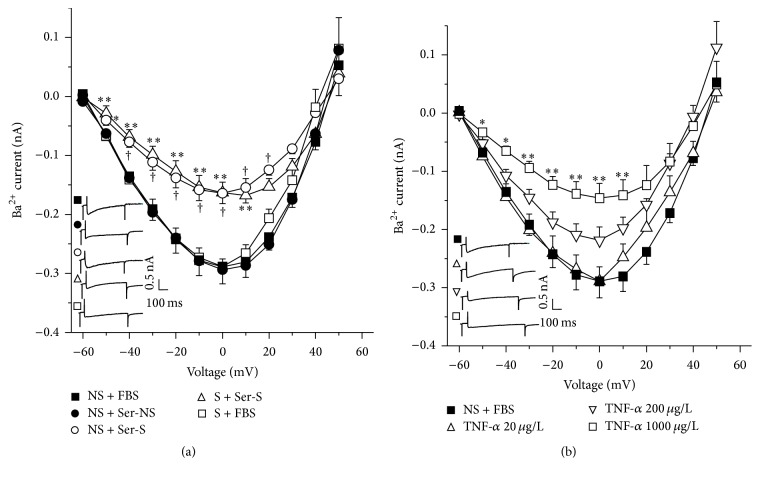
Voltage dependent L-type Ca^2+^ currents were diminished by sensitized guinea pig serum and by TNF-*α* in guinea pig tracheal myocytes. (a) Similar Ba^2+^ currents, equivalent to Ca^2+^ currents, induced by 10 mV increments were observed in myocytes from nonsensitized (NS) animals grown with fetal bovine serum (FBS) or serum from nonsensitized guinea pigs (Ser-NS). These currents were significantly diminished when myocytes from NS and sensitized (S) animals were grown with serum from sensitized guinea pigs (Ser-S, 10%). This phenomenon was not seen when myocytes from S were grown with FBS (*n* = 9 for each group). (b) Ba^2+^ currents in myocytes from NS grown with FBS showed a decrease when TNF-*α* was added. These responses were concentration dependent and only the highest (1000 *μ*g/L) produced a significant reduction of this current (*n* = 5 for each group). Insets in each figure represent original recordings. ^*∗*^ *p* < 0.05, ^*∗∗*^ *p* < 0.01, and ^†^ *p* < 0.01 when compared with NS + FBS group. Symbols represent mean ± SEM.

**Figure 3:**
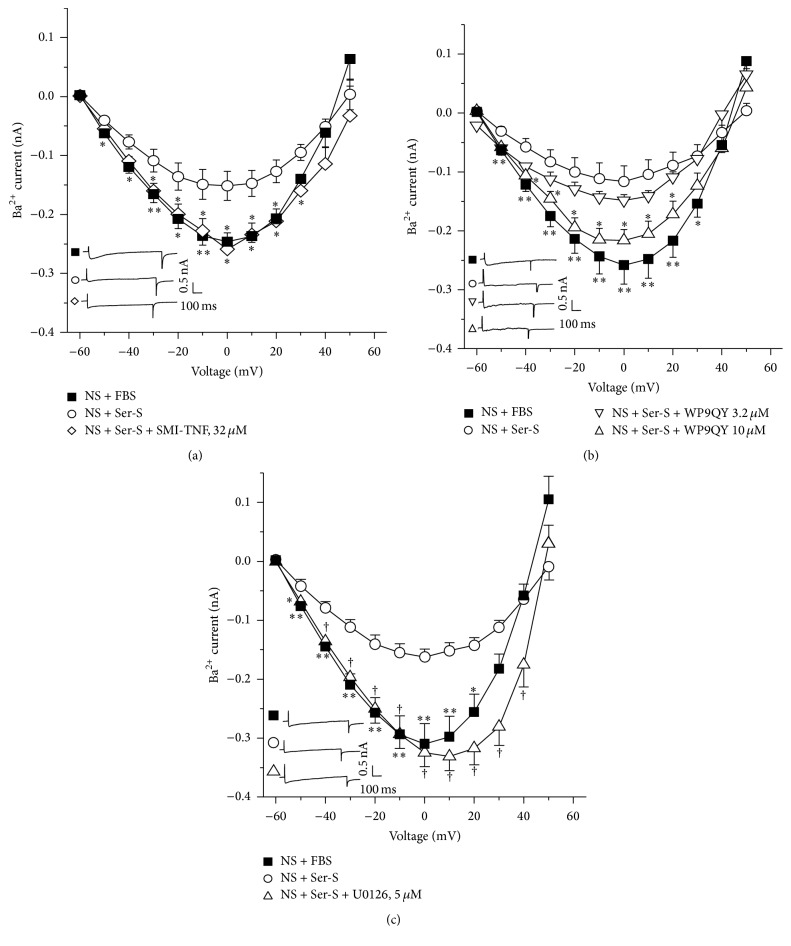
TNF-*α* diminishes the L-type Ca^2+^ currents through activation of the TNF-*α* receptor 1 (TNFR1) and MAP kinase signaling pathway in guinea pig tracheal myocytes. (a) Myocytes from nonsensitized animals (NS) grown with fetal bovine serum (FBS) showed an inward Ba^2+^ current. This current was reduced when NS myocytes were grown with serum from sensitized guinea pig (Ser-S, 10%). This decrease was abolished when the small-molecule inhibitor of TNF-*α* (SMI-TNF, *n* = 7), which impedes this cytokine from binding to its receptor, was incubated during cell growth. (b) The Ba^2+^ current diminution induced by Ser-S incubation in NS myocytes was also reversed, in a concentration dependent manner, by the TNFR1 antagonist (WP9QY, *n* = 9). (c) The Ba^2+^ current decrease induced by Ser-S was abolished when myocytes were incubated with an inhibitor of ERK 1/2 kinase (U0126, *n* = 6). Insets in each figure represent original recordings. ^*∗*^ *p* < 0.05, ^*∗∗*^ *p* < 0.01, ^†^ *p* < 0.01 when compared with NS + Ser-S group. Symbols represent mean ± SEM.

**Figure 4:**
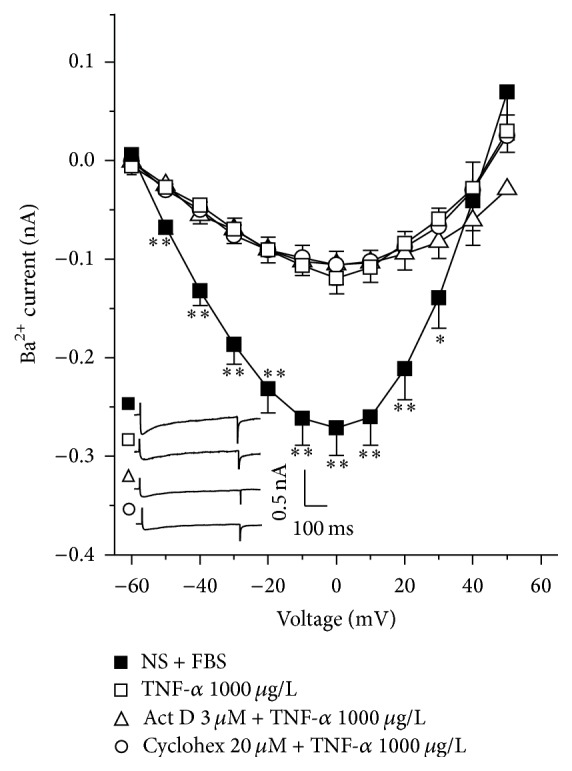
The Ba^2+^ current reduction induced by TNF-*α* is not mediated by a synthetic pathway activation in guinea pig tracheal myocytes. Ba^2+^ current evoked by step depolarization from −60 to 50 mV in tracheal cells from nonsensitized guinea pigs added with fetal bovine serum (NS + FBS, *n* = 7) were significantly diminished when myocytes were grown with TNF-*α* (*n* = 7). Neither actinomycin D (Act D, *n* = 6) nor cycloheximide (Cyclohex, *n* = 9) addition during myocyte growth altered TNF-*α* induced effect on the Ba^2+^ current. Inset represents original recordings. ^*∗*^ *p* < 0.05, ^*∗∗*^ *p* < 0.01, when compared with TNF-*α* group. Symbols represent mean ± SEM.

In the voltage clamp experiments with single myocytes from NS animals grown with fetal bovine serum (FBS), step depolarizations from −60 to 50 mV from a holding potential of −60 mV produced a voltage dependent inward Ca^2+^ current (ICa). The peak inward current reached maximal amplitude at 0 mV. This current was corroborated to be from the L-type Ca^2+^ channels, because it was almost abolished (86.35 ± 1.42%, data not shown) by 1 *μ*M nifedipine (*n* = 4), an L-type Ca^2+^ channel blocker. A similar ICa was observed in myocytes from NS guinea pigs cultivated with serum from NS animals (Ser-NS). When NS myocytes were grown with serum from sensitized guinea pigs (Ser-S) ICa was significantly diminished and this effect was also noticed in cells from S animals incubated with Ser-S. This last effect was not seen when S myocytes were cultured with FBS (Figure 2(a)). NS myocytes grown with different TNF-*α* concentrations showed a concentration dependent decrease of ICa, reaching only statistical significance to the highest concentration tested (1000 *μ*g/L, Figure 2(b)). These findings suggest that Ser-S contains some chemical mediator responsible for this ICa decrement, possibly TNF-*α*. This hypothesis was confirmed by using a small-molecule inhibitor of TNF-*α* (SMI-TNF) and a TNF-*α* receptor 1 (TNFR1) antagonist (WP9QY). We found that SMI-TNF completely abolished the ICa decrease induced by Ser-S in NS myocytes; in this experimental protocol, the current values observed were equal to those reached by the control group (NS + FBS, Figure 3(a)). In this regard, WP9QY showed a concentration dependent effect on the Ser-S induced ICa decrement and only the highest concentration tested reached statistical significance (10 *μ*M, Figure 3(b)). The exploration of TNFR1 signaling pathway by blocking the ERK 1/2 kinase with U0126, also showed a reversal of the decrease in ICa induced by Ser-S in NS myocytes (Figure 3(c)). Neither cycloheximide nor actinomycin D showed any effect on the TNF-*α*-induced ICa reduction, demonstrating that this response is not related to a synthetic pathway (Figure 4).

### 3.3. L-VDCC Subunits Ca_V_1.2 and Ca_V_1.3 Are Expressed in Guinea Pig Airway Smooth Muscle

**Figure 5:**
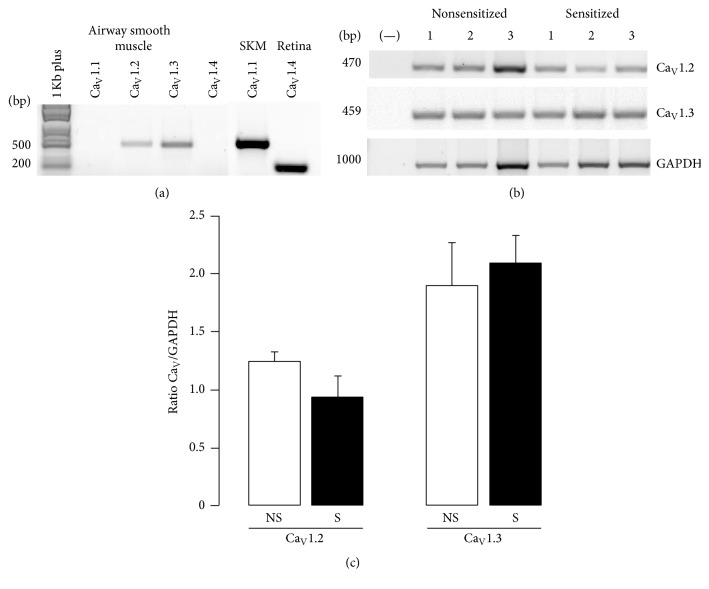
Detection of mRNA for L-VDCC subunits in guinea pig tracheal smooth muscle, as revealed by RT-PCR. (a) In airway smooth muscle, the PCR products at 470 and 459 bp length correspond to Ca_V_1.2 and Ca_V_1.3 cDNA, respectively. In this tissue, Ca_V_1.1 and Ca_V_1.4 were not found. Positive controls for these subunits were skeletal muscle (SKM, ~500 bp) and retina (~200 bp). Lane at the left corresponds to 1 Kb Plus DNA Ladder. (b) Representative PCR blots for Ca_V_1.2 and Ca_V_1.3 from nonsensitized (NS, *n* = 3) and sensitized (S, *n* = 4) smooth muscles. The first column in each blot corresponds to a negative control without template. The lower panel displays constitutive cDNA of GAPDH. (c) Densitometry data analysis for mRNA from Ca_V_1.2 and Ca_V_1.3 subunits showing no statistical significance between NS and S. Bars correspond to mean ± SEM.

**Figure 6 fig6:**
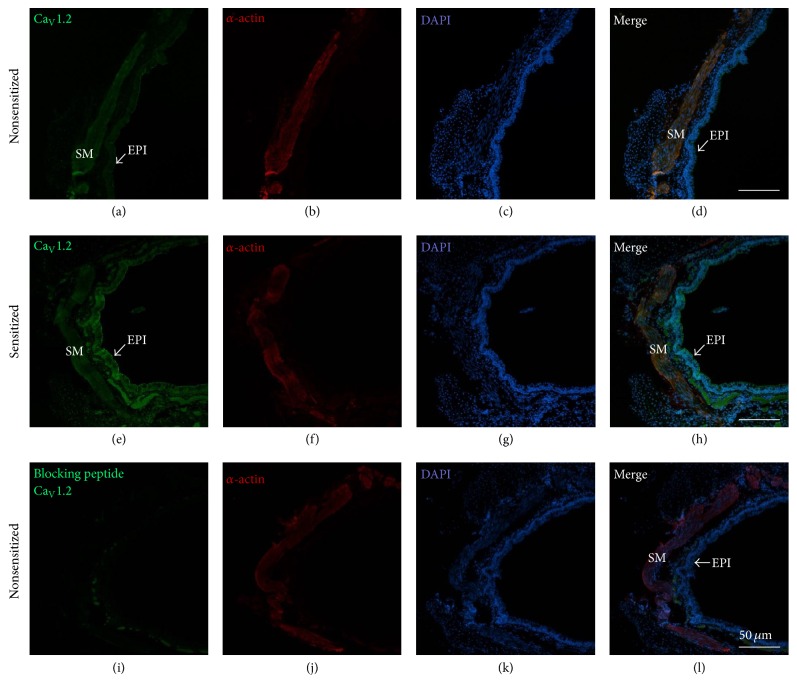
Immunofluorescence for Ca_V_1.2 in nonsensitized and sensitized guinea pig tracheal smooth muscle. The first column shows immunoreactivity for Ca_V_1.2 (stained green) in nonsensitized (a) and sensitized tissues (e); notice that Ca_V_1.2 is located in the airway smooth muscle (SM) and epithelium (EPI, pointed by arrow); blocking peptide completely eliminated the fluorescence (i). The second and the third columns illustrate smooth muscle *α*-actin (stained red; (b), (f), (j)) and cell nuclei (DAPI, stained blue; (c), (g), (k)). The last column depicts merged images of the former three columns ((d), (h), (l)). In these merged images, Ca_V_1.2 is seen to be colocalized with *α*-actin (stained yellow) on the smooth muscle.

**Figure 7:**
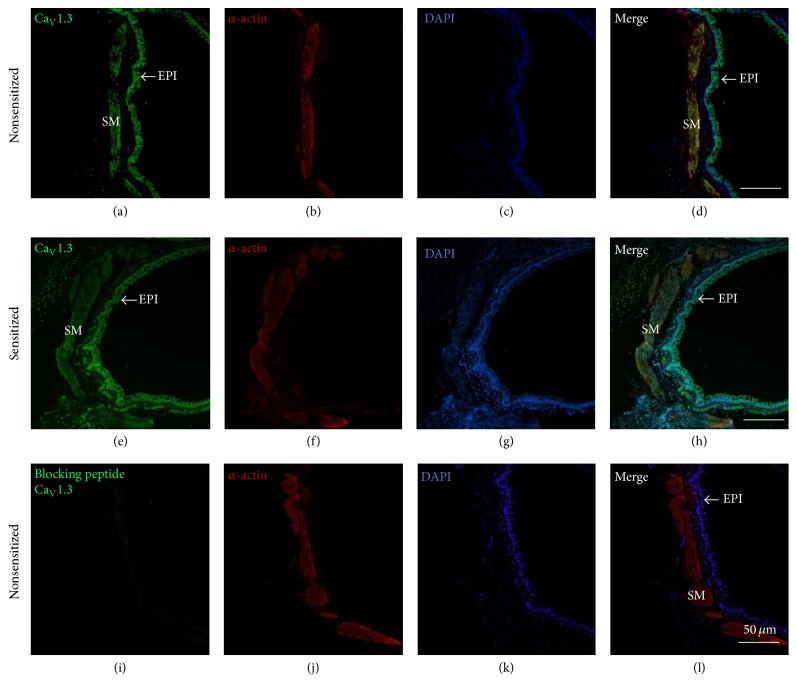
Immunofluorescence for Ca_V_1.3 in nonsensitized and sensitized guinea pig tracheal smooth muscle. The first column shows immunoreactivity for Ca_V_1.3 (stained green) in nonsensitized (a) and sensitized tissues (e); notice that Ca_V_1.3 is located in the airway smooth muscle (SM) and epithelium (EPI, pointed by arrows); blocking peptide completely eliminated the fluorescence (i). The second and the third columns illustrate smooth muscle *α*-actin (stained red; (b), (f), (j)) and cell nuclei (DAPI, stained blue; (c), (g), (k)). The last column depicts merged images of the former three columns ((d), (h), (l)). In these merged images, Ca_V_1.3 is seen to be colocalized with *α*-actin (stained yellow) on the smooth muscle.

In guinea pig airway smooth muscle from NS animals, we found that the main mRNA was for Ca_V_1.2 followed by Ca_V_1.3. The mRNA for Ca_V_1.1 and Ca_V_1.4 were not expressed in this tissue. Smooth muscle from S animals showed a similar expression pattern in the Ca_V_ subunits mRNA, and these results were not different from NS tissues (Figure 5). We corroborated that the primers used for Ca_V_1.1 and Ca_V_1.4 were adequate by testing them in positive control tissues: skeletal muscle for Ca_V_1.1 and retina for Ca_V_1.4 (Figure 5). Since the mRNA for Ca_V_1.2 and Ca_V_1.3 were the main subunits found in guinea pig airway smooth muscle, we performed immunofluorescence for Ca_V_1.2 and Ca_V_1.3 proteins in both NS and S tissues. We found that Ca_V_1.2 and Ca_V_1.3 are present in NS and S guinea pig airway smooth muscle and also in the epithelium and cilia. Negative controls carried out by the incubation of the respective blocking peptide showed no staining (Figures 6(i) and 7(i)).

## 4. Discussion

In the present study we found that S guinea pig tracheas had a lower contractile response to KCl when compared to NS tissues. This finding was mimicked when NS tracheas were incubated with serum from sensitized animals (Ser-S). Additionally, ICa from NS or S myocytes was reduced when cells were grown with Ser-S and this effect was not seen when S cells were grown with FBS pointing out that some inhibitory factor was present in the Ser-S. This ICa decrease was also observed when NS myocytes cultured with FBS were added with TNF-*α* (Figure 2(b)). We corroborated that the chemical mediator present in Ser-S was TNF-*α* because the SMI-TNF and the TNFR1 antagonist abolished the ICa decrease induced by Ser-S. Additionally, the ERK 1/2 kinase inhibitor also reversed the ICa reduction induced by Ser-S implying a MAP kinase-dependent pathway. Furthermore, the main subunits of the L-VDCC expressed in guinea pig airway smooth muscle were Ca_V_1.2 and Ca_V_1.3, and sensitization did not modify their expression.

We recently found that, in guinea pig airway smooth muscle, L-VDCC and store operated Ca^2+^ channels (SOC, capacitative Ca^2+^ entry) are the main membrane Ca^2+^ handling proteins involved in providing extracellular Ca^2+^ for SR Ca^2+^ refilling to sustain contraction [12]. Additionally, former works in porcine and human airway smooth muscle claimed that L-VDCC and receptor operated Ca^2+^ channels (ROC) were the main channels involved in this mechanism [30, 31]. In this context, each of these channels only partially mediates the sustained contraction, although L-VDCC seems to participate to a lesser extent than SOC. Interestingly, when both channels (L-VDCC and SOC) were consecutively blocked, a potentiation effect was seen [32].

The inflammatory condition developed by sensitization induced that, in guinea pig tracheas, KCl responses were notably diminished and this effect was reproduced by Ser-S. Because TNF-*α* concentration has been demonstrated to be increased in plasma and bronchoalveolar lavage fluid from sensitized guinea pigs [6], we hypothesise that this cytokine was responsible for this decreased response. This was confirmed when TNF-*α* diminished ICa in single NS tracheal myocytes in the same way as Ser-S (Figure 2). These findings seem paradoxical, since it is recognized that TNF-*α*, a proinflammatory cytokine, alters normal airway myocytes to a hyperreactive state. In this context, in human airway smooth muscle, this cytokine induces an increment in the capacitative Ca^2+^ entry due to an increased expression of STIM1 an Orai1 [33, 34]. Furthermore, in this tissue TNF-*α* also causes an upregulation of CD38 expression, a cell surface protein that regulates the synthesis and degradation of cyclic ADP-ribose (cADPR) [35]. This molecule provokes Ca^2+^ release from the SR through stimulation of the ryanodine receptor. The overexpression of CD38 could augment cADPR and SR Ca^2+^ release favoring airway hyperresponsiveness. Moreover, the sarcoplasmic reticulum ATPase (SERCA) expression in human airway smooth muscle exposed to TNF-*α* was decreased [36]. All the abovementioned evidences point out that TNF-*α* augments cytosolic Ca^2+^ to promote airway hyperresponsiveness. Because these mechanisms that increase cytosolic Ca^2+^ concentration are upgraded by TNF-*α*, the need for further Ca^2+^ entry through L-VDCC could be less; therefore it seems conceivable that this cytokine might induce a reduced ICa as compensatory effect.

We found that the subunits of L-VDCC in guinea pig tracheal smooth muscle were Ca_V_1.2 and Ca_V_1.3 and that their expression was not modified by sensitization. Thus, the ICa observed in this tissue mainly corresponds to these subunits. Therefore, the ICa decrease induced by TNF-*α* was related to L-VDCC function and not to a reduction in their expression during sensitization (Figures 5 [Fig fig6]–7).

It is well known that TNF-*α* receptors are coupled to a mitogen-activated protein (MAP) kinases cascade involving either ERK, JNK, or p38 MAPKs to induce several transcription factors that control gene expression [37]. This MAP kinase signaling pathway usually induces gene expression in nonmuscle cells, while, additional to gene expression in airway smooth muscle cells, muscarinic M_2_ receptor stimulation leads to caldesmon phosphorylation through ERK MAP kinases [38]; when it is in its nonphosphorylated state, caldesmon inhibits the actomyosin ATPase and reduces smooth muscle force production [39]. Thus, TNF-*α* effect on L-VDCC in our tissue could be due either to a phosphorylation mechanism or to a synthetic pathway.

**Figure 8:**
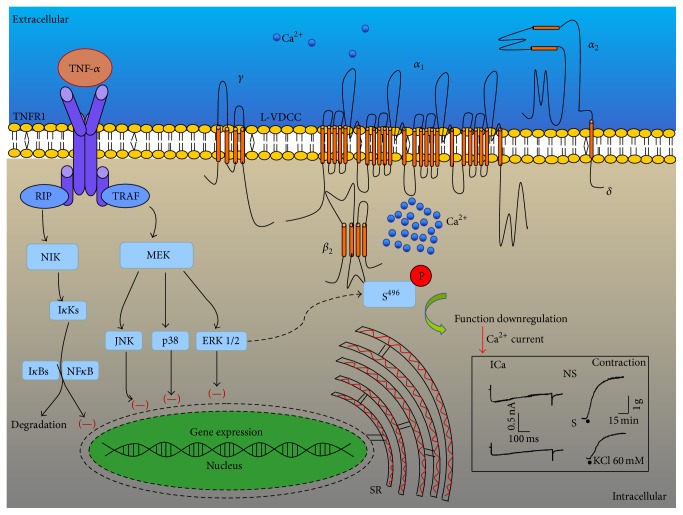
Schematic representation of the proposed mode of action of tumor necrosis factor *α* (TNF-*α*) on the L-type voltage dependent calcium channel (L-VDCC) of the guinea pig airway smooth muscle. TNF-*α* whether exogenous or present in sensitized guinea pig serum (Ser-S) activates its receptor 1 (TNFR1). Afterwards, it promotes synthetic signaling pathways: it activates receptor interacting protein (RIP), NF-*κ*B-inducing kinase (NIK), and I*κ*B kinases (I*κ*Ks) that phosphorylate NF*κ*B inhibitors (I*κ*Bs) activating nuclear factor *κ*B (NF*κ*B) and through TNF receptor-associated factor (TRAF), map kinase kinase (MEK) and extracellular signal-regulated kinase (ERK 1/2), p38 MAPK, or c-Jun N-terminal kinase (JNK). We demonstrated that a synthetic pathway was not responsible for the diminution in the Ca^2+^ current (ICa, see Figure 4). Nevertheless, ERK 1/2 might be directly phosphorylating serine^496^  (S^496^) on the *β*_2_ subunit of the L-VDCC, favoring a downregulation of the ICa. Inset illustrates ICa in tracheal myocytes and contraction in tracheal rings from guinea pigs. NS implies nonsensitized tissues or cells and this indicates the absence of exogenous TNF-*α* or serum from Ser-S. S illustrates original recordings from sensitized tissues or cells in the presence of Ser-S; notice that both ICa and contraction are diminished. Other subunits of the L-VDCC: *α*_1_, *α*_2_, *γ*, *δ*. SR: sarcoplasmic reticulum.

In airway smooth muscle TNF-*α* exerts its actions by activating TNFR1 and TNFR2 receptors. At least the upregulation of CD38 expression induced by this cytokine has been confirmed to be mediated by TNFR1 activation of downstream ERK and p38 MAP kinase signaling pathway without involving NF-*κ*B nor AP-1 nuclear transcription factors [18]. In NS myocytes from guinea pig tracheas, the presence of Ser-S induced an ICa reduction that was abolished by a small-molecule inhibitor of TNF-*α*, confirming that this cytokine is responsible for this effect; TNF-*α* induced the same ICa decrease as Ser-S. Furthermore, we corroborated that the receptor involved in this ICa reduction was TNFR1, because WP9QY, an antagonist of this receptor, reversed the ICa diminution. Therefore, we verified that ERK signaling pathway was involved in this ICa reduction, since U0126 completely abolished this effect. Because this signaling pathway usually turns on transcription factors and therefore protein synthesis, we confirmed that this was not the case; neither actinomycin, a transcription inhibitor, nor cycloheximide, a protein synthesis inhibitor, had any effect on the ICa decrease induced by TNF-*α*. Thus, our results point out that this cytokine effect is probably related to a phosphorylation process of the L-VDCC through ERK 1/2 MAP kinase. In this regard, ERK 1/2 MAP kinase has been implicated in phosphorylating rat ventricular myocytes L-VDCC in two sites: *β*_2_ Ser^496^ and *α*_1_ Ser^1928^. Phosphorylation of the former site may be linked to downregulation of the L-VDCC activity, while the second site's phosphorylation may lead to upregulation of the function [40]. Therefore, TNF-*α* activation of MAP kinase pathway may be phosphorylating the L-VDCC in *β*_2_ Ser^496^ to reduce the ICa in guinea pig airway smooth muscle (Figure 8), although further research is required.

## Figures and Tables

**Table 1:** Sequence of primers and PCR conditions for the different subunits of L-VDCC.

Gene	Oligonucleotides 5′-3′	Length (bp)	Amplification conditions	Reference
Ca_V_1.1	Fw: TGGTACGTCGTCACCTCCT	237	MT = 56°C	XM_013158049
	Rv: CATCTATGATGCTGCCGATG		34 cycles	

Ca_V_1.2	Fw: AATTGCTCTGAAGATGACAGC	471	MT = 56°C	NM_001172923.1
	Rv: AGCTGCCAGGACATTGTCG		30 cycles	

Ca_V_1.3	Fw: TCCCGCCGGCAGGACTAT	459	MT = 56°C	XM_005008263
	Rv: ATCACCTTTAACCTCCCCCCA		32 cycles	

Ca_V_1.4	Fw: TACCCATCCCGGGTACCTAT	436	MT = 56°C	XM_013144287
	Rv: GAAGTGGGAGAAGATAGACT		34 cycles	

GAPDH	Fw: TGAAGGTGAAGGTCGGTGTGAACG Rv: CATGTAGGCCATGAGGTCCACCAC	~1000	MT = 56°C 29 cycles	Chávez et al., 2013 [29]

Fw: forward primer, Rv: reverse primer, MT: melting temperature, and bp: base pairs.

## Abbreviations

TNF-*α*: Tumor necrosis factor alpha
L-VDCC: L-type voltage dependent calcium channel
Ser-S: Serum from sensitized animals
NS: Nonsensitized guinea pig
S: Sensitized guinea pig
ICa: Voltage dependent inward calcium current
SMI-TNF: Small-molecule inhibitor of TNF alpha
TNFR1: Tumor necrosis factor receptor 1
WP9QY: TNF alpha receptor 1 antagonist
Ca_V_1.2: Voltage dependent calcium channel 1.2
Ca_V_1.3: Voltage dependent calcium channel 1.3
mRNA: Messenger ribonucleic acid
ERK 1/2: Extracellular signal-regulated kinases
SR: Sarcoplasmic reticulum
TNFR2: Tumor necrosis factor receptor 2
CD38: Cluster of differentiation 38
cADPR: Cyclic adenosine diphosphate ribose
p38 MAPKs: p38 mitogen-activated protein kinases
JNK: Jun NH_2_-terminal kinase
MAPK: Mitogen-activated protein kinases
Ca_V_1.1: Voltage dependent calcium channel 1.1
Ca_V_1.4: Voltage dependent calcium channel 1.4
i.p.: Intraperitoneal
s.c.: Subcutaneous
OA: Ovalbumin
FBS: Fetal bovine serum
Ser-NS: Serum from nonsensitized animals
SMI-TNF: Small-molecule inhibitor of TNF alpha
U0126: 1,4-Diamino-2,3-dicyano-1,4-bis-(0-amino-phenylmercapto)butadiene ethanolate, inhibitor of MEK
MT: Melting temperature
PBS: Sodium phosphate buffer
BP: Blocking peptide
EPI: Epithelium
SM: Smooth muscle
SOC: Store operated calcium channels
ROC: Receptor operated calcium channel
STIM: Stromal interaction molecule
SERCA: Sarcoplasmic reticulum calcium ATPase
NF-*κ*B: Nuclear factor kappa B
AP-1: Activator protein
S^496^: Serine^496^
NIK: NF-kappaB-inducing kinase
I*κ*Ks: I kappa B kinases
MEK: Mitogen-activated protein kinase kinase
TRAF: TNF receptor-associated factor
I*κ*Bs: NF-kappa B inhibitors
RIP: Receptor interacting protein.
